# Case Report: Severe brigatinib-induced pneumonitis in a patient with EML4–ALK+ metastatic non-small cell lung adenocarcinoma

**DOI:** 10.3389/fonc.2025.1572425

**Published:** 2025-04-24

**Authors:** Khyati Somayaji Dasika, Megan Melody, Jyoti D. Patel

**Affiliations:** ^1^ Department of Medicine, Northwestern University, Chicago, IL, United States; ^2^ Division of Hematology/Oncology, Tampa General Hospital Cancer Institute, Tampa, FL, United States; ^3^ Department of Medicine, Division of Medical Oncology, Northwestern University, Chicago, IL, United States

**Keywords:** NSCLC, pneumonitis, tyrosine kinase inhibitor (TKI), steroids, case report

## Abstract

Anaplastic lymphoma kinase (ALK) tyrosine kinase inhibitors (TKIs), such as brigatinib, are targeted therapies for metastatic non-small cell lung cancer (mNSCLC). We present a patient with echinoderm microtubule-associated protein-like 4 (*EML4*) and *ALK* fusion protein-positive mNSCLC who developed severe hypoxemia and pneumonitis requiring intubation within 2 days of initiating brigatinib therapy. The workup for alternative etiologies of respiratory distress was unrevealing, and the patient was treated for presumed brigatinib-induced pneumonitis with high-dose methylprednisolone. This case demonstrates high-grade, rapidly progressive brigatinib-induced pneumonitis with prompt clinical improvement after steroids and a marked disease response without recurring toxicity after treatment with an alternative ALK TKI, alectinib.

## Introduction

Anaplastic lymphoma kinase (ALK) tyrosine kinase inhibitors (TKIs) have emerged in the last decade as efficacious front-line treatments for ALK+ metastatic non-small cell lung cancer (mNSCLC). They function by inhibiting the receptor tyrosine kinase, halting a key step in cancer cell growth and differentiation (1). Brigatinib is a second-generation ALK TKI developed in 2017 to address resistance mutations associated with first-generation ALK TKIs, including the “gatekeeper mutation” in the kinase domain of ALK and the notably recalcitrant G1202R mutation (2, 3). Brigatinib achieves robust progression-free survival in patients with ALK+ mNSCLC, particularly with intracranial involvement and when utilized in the first-line setting (3, 4).

Despite their efficacy in the treatment of non-small cell lung cancer (NSCLC), ALK TKIs have a broad toxicity profile including shortness of breath, gastrointestinal (GI) intolerance, anemia, elevated liver enzymes, and fatigue (5). Although the toxicity profile is relatively broad, the severity of these toxicities is generally mild and usually does not necessitate the discontinuation of ALK TKI treatment. For example, a meta-analysis of 4,177 patients reported approximately 40%–50% of GI-related side effects such as nausea, vomiting, diarrhea, and constipation, with only 2.5% high-grade toxicities (grade 3 or 4) requiring treatment termination (6). Pulmonary toxicities requiring treatment termination, including interstitial lung disease, pneumonitis, and pulmonary fibrosis, are also relatively rare, occurring in approximately 0.2%–10.9% of the total patients with ALK+ mNSCLC (7). Of all pulmonary toxicities, pneumonitis is one of the most common and could potentially be fatal if not addressed promptly. It is mediated by ALK TKI-induced cellular destruction of the alveolar epithelium and subsequent tissue inflammation (8).

Brigatinib is associated with the highest incidence of pulmonary toxicities occurring within the first few days after therapy initiation, much earlier in the treatment course when compared with alternative ALK TKIs such as alectinib and crizotinib (9, 10). These toxicities are generally mild, self-limited, and relatively rare, especially with the implementation of a step-up dosing of brigatinib, a gradual escalation of dosage from 90 to 180 mg over several days (11). Moreover, clinical risk factors such as older age and smoking status are generally associated with an increased incidence of pneumonitis in patients with mNSCLC (9). In a meta-analysis of 4,752 patients with advanced-stage NSCLC treated with various ALK TKIs, subgroup analysis of the brigatinib-treated patients demonstrated a 7% incidence of all-grade pneumonitis and a 3% incidence of high-grade pneumonitis (10). The overall incidence of all-grade and high-grade pneumonitis for all other ALK TKIs was much lower, demonstrating the relatively higher toxicity of brigatinib (10). Although the exact pathogenesis of brigatinib-induced pneumonitis is unknown, one proposed mechanism is cytokine-mediated inflammatory reactions in the alveolar capillary walls leading to an altered membrane permeability and capillary fluid leakage. Other suggested mechanisms of brigatinib-induced lung toxicity include immune-mediated inflammation of the lung tissue or a direct cytotoxic effect on the lung parenchyma (12).

Although brigatinib is the ALK TKI most strongly associated with pneumonitis, this case underscores a novel phenomenon concerning the swift onset of and recovery from brigatinib-induced pneumonitis, an aspect not previously explored in the literature. We demonstrate a case of severe, rapidly progressive brigatinib-induced pneumonitis requiring escalation of care to the intensive care unit (ICU) and subsequent intubation less than 2 days after brigatinib initiation. Existing case reports have discussed pulmonary toxicities of epidermal growth factor receptor (EGFR) and mesenchymal epithelial transition (MET) TKIs. However, we uniquely present an example of a rapid-onset, high-grade pneumonitis from an ALK-specific TKI (13, 14). This case also highlights the relatively rapid reversibility of pneumonitis with steroid treatment and cessation of brigatinib together with a successful rechallenge with an alternative ALK TKI, achieving disease response without additional adverse effects.

## Case description

**Figure 1:**
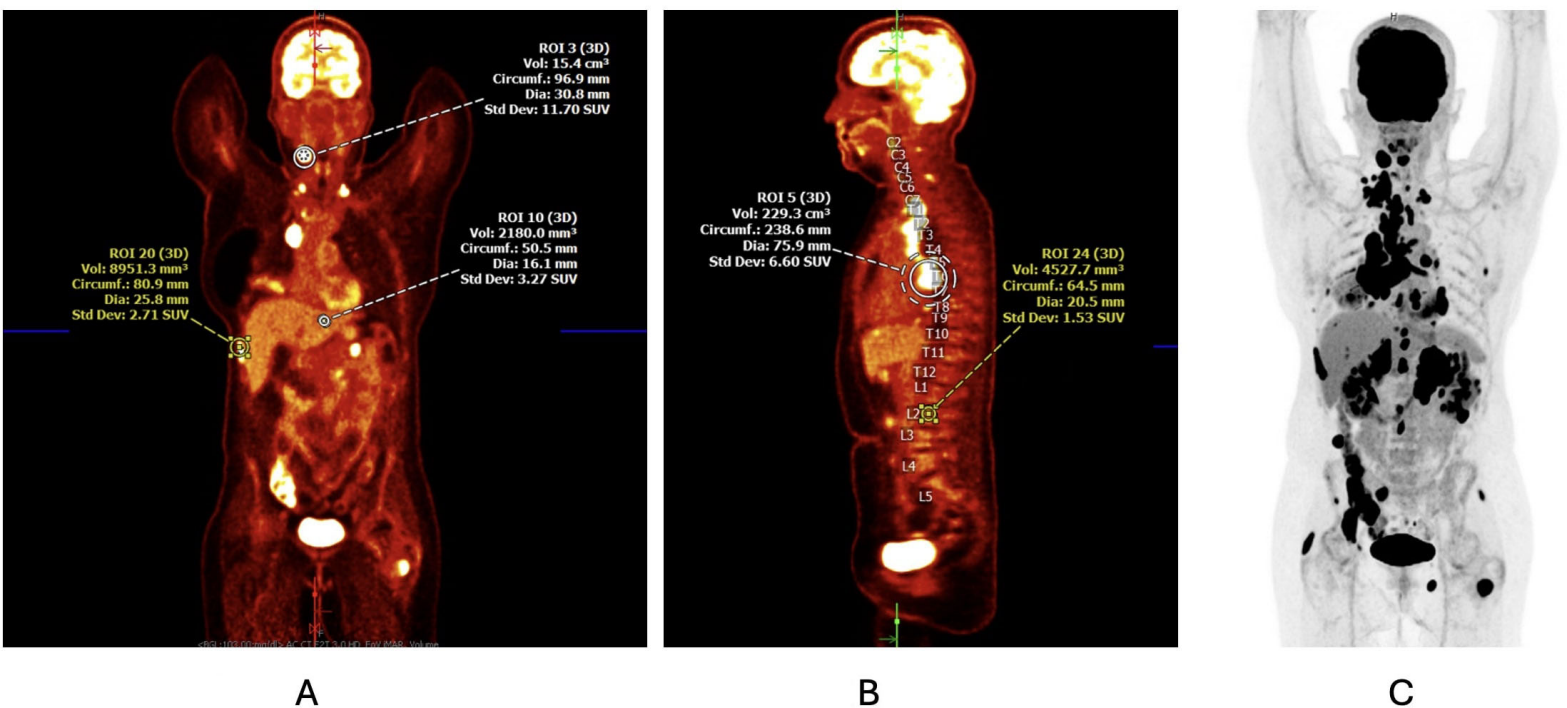
**(A, B)** Initial PET/CT scan at the time of diagnosis demonstrating a 6.4-cm × 4.8-cm × 7.0-cm soft tissue mass in the right perihilar region with resultant complete occlusion of the right middle lobe bronchus and right lower lobe ground-glass opacity and atelectasis. Mediastinal, paratracheal, paraesophageal, subcarinal, perihilar, and supraclavicular lymphadenopathy along with known osseous metastasis is also noted. **(C)** Corresponding PET scan image detailing the areas of cellular activity.

We present a 58-year-old woman with a history of 15 pack-year tobacco use and a newly diagnosed mNSCLC with metastases to the liver and adrenal glands and widespread osseous disease in the calvarium with extension into the underlying dura (**Figures 1A–C**). Immunohistochemistry showed programmed cell death protein ligand 1 (PD-L1) positivity in 55% of tumor cells, and molecular analysis with a next-generation sequencing assay was positive for an *EML4* and *ALK* gene fusion. A right supraclavicular lymph node biopsy confirmed lung adenocarcinoma. Before the patient could establish care with an outpatient oncologist, she presented to the emergency department 2 weeks after the initial diagnosis with intractable abdominal and flank pain. CT scan of the chest, abdomen, and pelvis showed increased size of the primary right perihilar pulmonary tumor, bilateral compressive atelectasis, and a worsening hepatic and adrenal metastatic disease. She developed new-onset hypoxemia requiring 1–2 L of oxygen via a nasal cannula (NC), which was attributed to progressive NSCLC disease burden. MRI of the brain demonstrated a worsening osseous disease, while MRI of the cervical, thoracic, and lumber spine was concerning for leptomeningeal disease. Given the significant burden of symptomatic disease, hypoxia, and the presence of a targetable mutation ALK, her oncology team decided against delaying treatment to the outpatient setting. The patient was initiated on brigatinib 90 mg daily during her inpatient admission, with plan to increase the dosing to 180 mg daily after 1 week of therapy. Brigatinib was chosen among other first-line second-generation ALK TKIs, such as alectinib, ensartinib, and lorlatinib, given the patient’s high burden of intracranial disease and the more superior intracranial efficacy of brigatinib, particularly in the first-line setting (4). Furthermore, the broader range of activity of brigatinib against resistant mutations and the ability to use a step-up dosing to conservatively manage side effects were initially prioritized (11).

**Figure 2:**
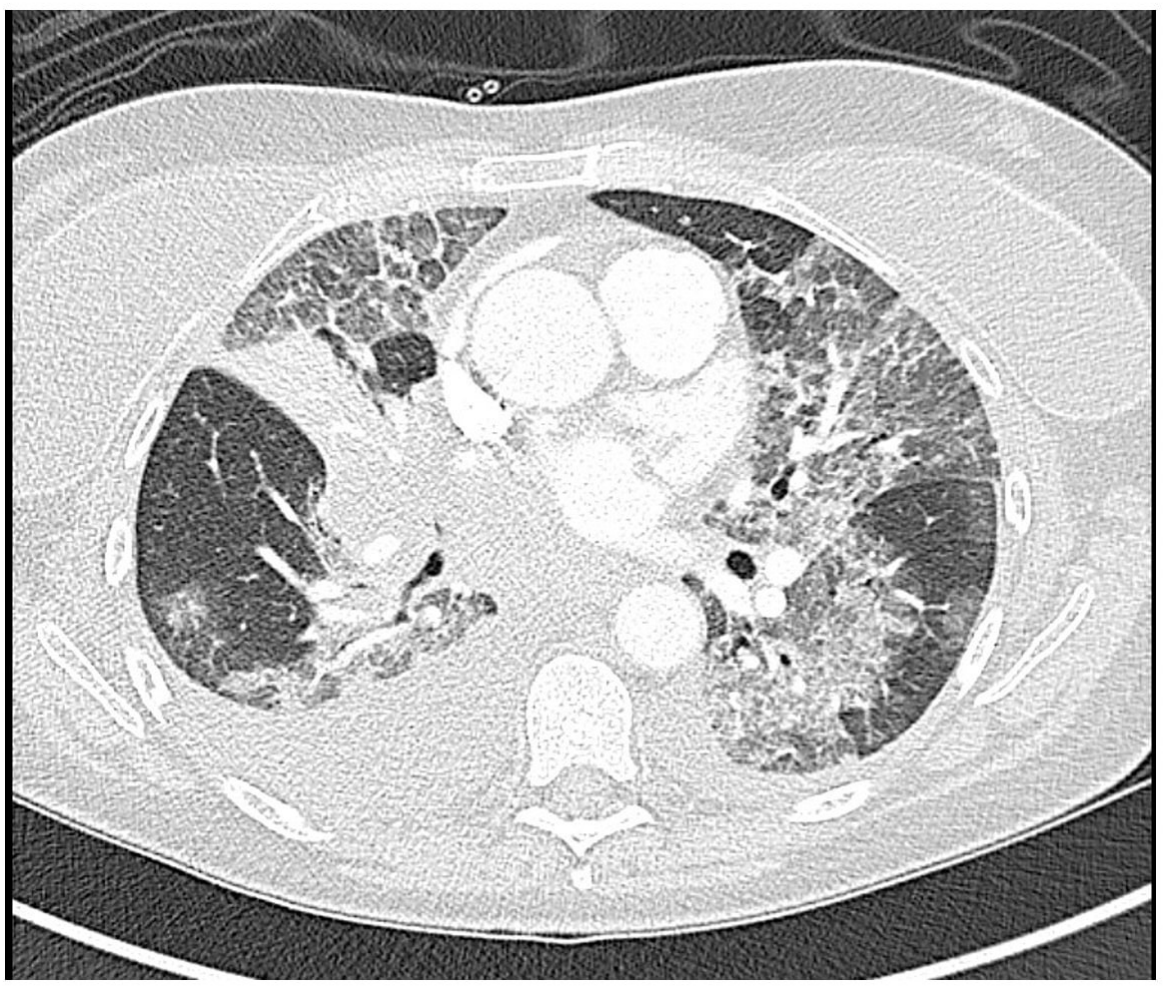
CT angiogram of the chest 38 h after initiation of brigatinib showing extensive bilateral ground-glass and reticular opacities along with interlobular septal thickening. There is also bilateral lower lobe atelectasis, new complete right middle lung atelectasis, and new right pleural effusion.

Within 8 h of the first dose of brigatinib, the patient developed a worsening dry cough and an increased oxygen requirement to 4 L NC. Physical exam was significant for rales throughout all lung fields. Chest X-ray demonstrated bilateral multifocal airspace disease and right-sided pleural effusion. CT angiogram of the chest was without pulmonary embolism, but showed new extensive ground-glass and reticular opacities not present on the initial admission CT scan from the emergency department, with highest concern for drug-induced pneumonitis and redemonstration of the right pleural effusion (**Figure 2**). She was immediately started on low-dose dexamethasone, empiric cefepime, and diuresis for management of pneumonia and pleural effusion *versus* a concern for brigatinib-induced pneumonitis. Given the relatively mild hypoxemia and the high NSCLC disease burden, treatment with brigatinib was continued. After 14 h of the second dose of brigatinib, the patient developed progressive hypoxemia with escalating oxygen requirements, eventually requiring 60 L/100% FiO_2_ on high-flow nasal canula (HFNC) to maintain SpO_2_ >90% and transfer to the ICU for higher level of care.

At the time of acute clinical change and transfer, the laboratory studies were notable for a leukocytosis of 22.5 × 10^9^/L, elevated brain natriuretic peptide (BNP) of 225 pg/ml, procalcitonin of 0.117 ng/ml, sedimentation rate of 122 mm/h, and C-reactive protein of 163 mg/L. Arterial blood gas showed a pH of 7.42, pCO_2_ of 35, and pO_2_ of 65, notable for hypoxemia. The patient eventually required intubation for respiratory muscle weakness and subsequent worsening hypoxia 3 days after the initiation of brigatinib.

Infectious workup including respiratory pathogen panel, sputum culture, *Legionella*, and *Streptococcus pneumoniae* urine antigens showed no abnormality. An empiric course of antibiotics did not improve the respiratory status of the patient. Furthermore, a course of diuresis did not improve her oxygen requirement, making hypervolemia-mediated cardiogenic pulmonary edema a less likely etiology of the hypoxemia. A right-sided chest tube was placed for the unilateral pleural effusion with 900 ml of transudative fluid output, without significant improvement in respiratory status. Cytology and pleural fluid studies were negative for infection and malignancy.

Given the onset of symptoms within 48 h after the initiation of brigatinib therapy, in conjunction with the imaging findings and no resolution with other therapies, suspicion for brigatinib-induced pneumonitis as the etiology of the patient’s hypoxemia and respiratory failure was high. In addition, CT scan of the chest showed extensive ground-glass opacities, reticular opacities, and interlobular septal thickening—characteristic radiographic findings of drug-induced pneumonitis. Brigatinib therapy was discontinued after two doses of 90 mg, and no further doses were administered after the patient’s acutely worsening hypoxia requiring transfer to the ICU. The patient was treated with intravenous (IV) methylprednisolone 1 mg/kg (60 mg) daily for 3 days, along with prophylactic antibiotics and diuresis. There are no established guidelines for steroid treatment for brigatinib-induced pneumonitis; therefore, we employed a similar approach used for other medication-induced pneumonitis. Typically, patients receive 1–4 mg kg^−1^ day^−1^ of IV steroids (i.e., methylprednisolone) for high-grade pneumonitis, starting on the lower end of the range and escalating as needed in the first 2–3 days (15). A thorough review of the patient’s pre-admission and current medications also revealed no interactions with brigatinib that could have caused an increased risk of severe pneumonitis.

The respiratory status of the patient improved 4 days after the initiation of high-dose steroids and was successfully extubated to 30 L HFNC and later transitioned to 2 L NC within hours. Within a 24-h period, supplemental oxygen was completely discontinued, and she maintained SpO_2_ >98%–99%, suggesting that steroids were necessary to reverse hypoxemia and pneumonitis. She started a prolonged prednisone taper with a 10-mg dose reduction every 7 days. Other immunomodulatory therapies such as infliximab and intravenous immunoglobulin (IVIG) have been sparingly used, but were not considered for our patient given her robust hypoxia improvement after IV methylprednisolone and the subsequent plan for a long-term oral steroid taper. Due to severe pulmonary toxicity, brigatinib was permanently discontinued, and alectinib 450 mg twice daily was started 3 days after extubation. She tolerated alectinib well, with no immediate adverse effects, and was discharged 9 days after extubation without needing supplemental oxygen.

**Figure 3:**
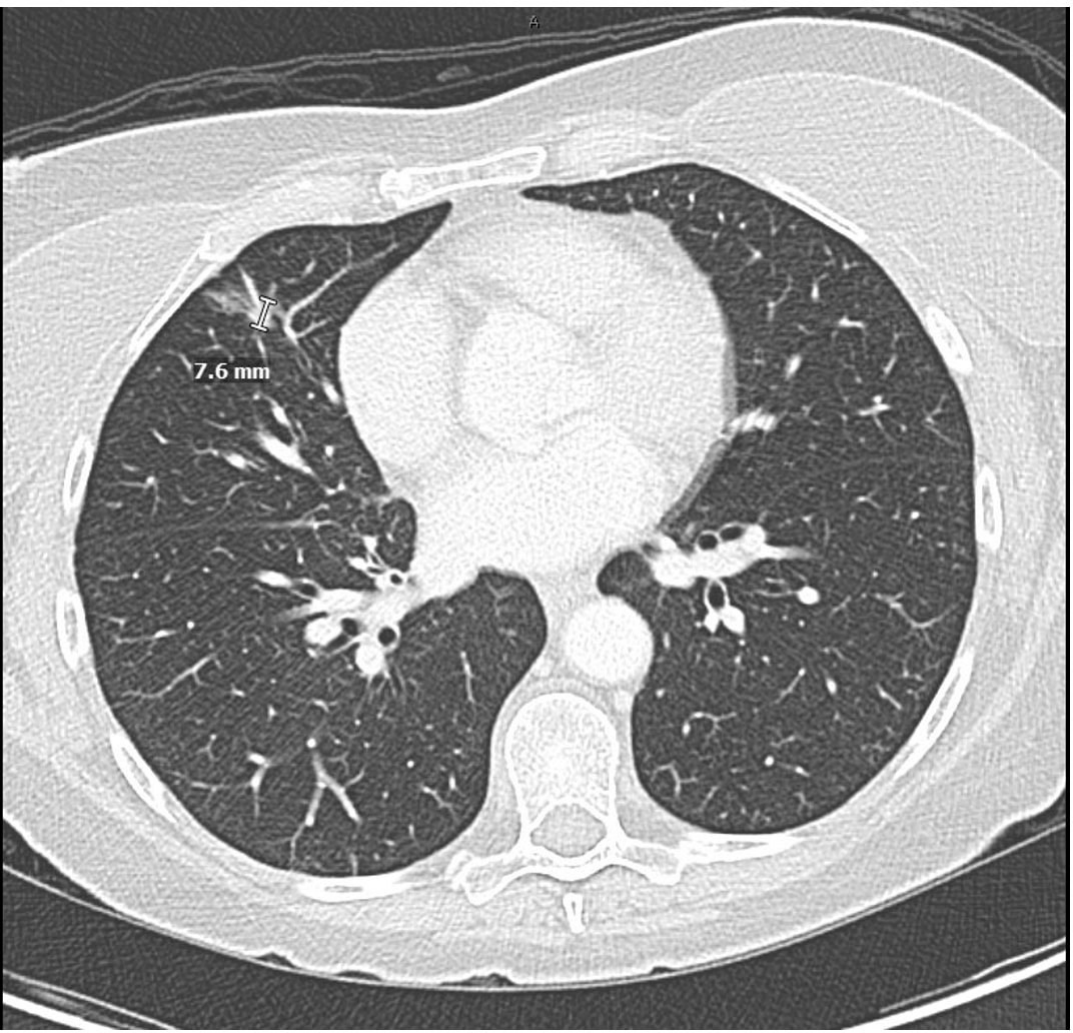
Chest CT with intravenous contrast 5 weeks after cessation of brigatinib demonstrating near-complete resolution of pneumonitis and resolution of right pleural effusion. Treatment response after 4 weeks of alectinib therapy showing decreased size of the primary right perihilar mass, decreased mediastinal and hilar lymphadenopathy, and improvement in metastatic disease.

At 2 weeks after discharge from the hospital, the patient presented to the oncology clinic and continued to tolerate alectinib therapy with no need for supplemental oxygen. She had no residual pulmonary complications such as chest tightness, shortness of breath, and cough. Her Eastern Cooperative Oncology Group (ECOG) performance status score was 1. She is restricted in strenuous exercises, but able to carry out light housework. The chest, abdomen, and pelvis CT scan obtained 5 weeks after brigatinib discontinuation showed near-complete resolution of the bilateral multifocal airspace disease (**Figure 3**). The mNSCLC disease burden decreased with smaller primary tumor, mediastinal lymph nodes, and hilar lymph nodes along with stable hepatic, adrenal, and osseous metastases. MRI of the brain demonstrated a decrease in the nodular areas of dural thickening, consistent with disease response with alectinib. Given the marked improvement in disease burden, the patient continued alectinib therapy.

**Figure 4:**
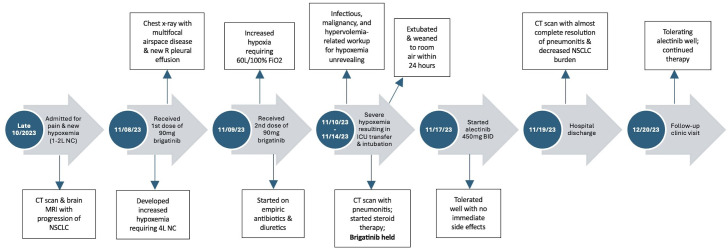
Timeline of the patient’s presentation, including the evolution of brigatinib-induced pneumonitis with subsequent resolution and future outcomes.

Subsequent CT scans 4 months after alectinib initiation showed a stable, sustained treatment response. The patient endorsed constipation from alectinib, but denied other side effects such as respiratory symptoms, nausea, myalgia, and leg swelling. Her lipid panel, thyroid hormone levels, kidney function, and hemoglobin A1c at the time were all within the normal limits. The patient underwent close monitoring of immunosuppressive effects such as infectious symptoms, high blood pressure, and metabolic derangements, with monthly clinic appointments or telemedicine visits in the first 3 months after hospital discharge. At 4 months post-initiation of alectinib therapy, she continued to note improvements in the respiratory status and performance status and now able to carry out all pre-deterioration activities. Her ECOG score was 0, and she is an avid traveler and hiker. She reported approaching strenuous activity more cautiously, but does not have breathing difficulties, is without activity limitations, and endorses a satisfactory, improved quality of life compared with the immediate post-hospital discharge period. Currently, the patient has been on alectinib for 6 months, without serious pulmonary adverse effects or progression of NSCLC. The patient's full clinical course is outlined in **Figure 4**.

## Discussion

This case highlights the acute onset of high-grade pneumonitis following first-time brigatinib use. The briskly evolving hypoxemia after brigatinib administration and the severity of pulmonary damage requiring intubation in the ICU juxtaposed with the rapid reversibility of the insult with steroid treatment and cessation of brigatinib are noteworthy. Moreover, our patient was transitioned to alectinib, another ALK TKI, without recurrence of pneumonitis and with continued NSCLC disease response.

For this patient, the rapid-onset pneumonitis following brigatinib initiation is more consistent with the direct cytotoxic effect on pulmonary tissues as the mechanism of brigatinib-induced pneumonitis. Immune-mediated inflammatory reactions typically develop over a longer period. Injury to the alveolar epithelium is followed by the release of cytokines and the recruitment of inflammatory cells, culminating in capillary leakage, pulmonary edema, and severe tissue inflammation (12).

The risk factors for high-grade pneumonitis after ALK TKI use include prior smoking history, concomitant medications, older age (>64 years), and renal dysfunction (creatinine clearance <80 ml/min) (16–18). In a retrospective analysis of 250 patients with ALK TKI-related pneumonitis, smoking history increased the risk of pneumonitis 3.61-fold (16). Increased age and decreased renal function can cause derangements in the metabolism of TKIs, contributing to drug accumulation and toxicity, while concurrent use of medications such as proton pump inhibitors, amlodipine, and magnesium oxide also increased the risk of pneumonitis compared with TKI monotherapy (17, 18). Our patient had a remote 15-pack-year smoking history, but no prior pulmonary conditions or significant risk factors, including concomitant use of proton pump inhibitors, amlodipine, magnesium oxide, and antifungals. Given the absence of preexisting lung disease aside from NSCLC, the severity and the rapid onset of pneumonitis were unexpected. Although the higher risk of pulmonary toxicity from brigatinib was considered prior to administration, it was difficult to predict the severity of toxicity. It is important to remain vigilant about pneumonitis in patients with one or more risk factors as a predictive model for the severity of brigatinib-induced pneumonitis. Furthermore, the lack of pretreatment pulmonary function assessment may have missed underlying abnormalities before brigatinib initiation. Evaluation of the baseline pulmonary function is crucial for future patients receiving medications with a high risk of early pulmonary adverse effects.

Step-up dosing of brigatinib with 90 mg for the first 7 days followed by 180 mg thereafter is used to mitigate early-onset pulmonary adverse events (19). Our patient developed pneumonitis after the second dose of brigatinib in a span of 2 days, highlighting a unique case of a rapid-onset pulmonary event compared with previous cases demonstrating the onset of pneumonitis from 4 to 5 days at the earliest (20). Historically, pulmonary adverse events have been associated with the higher dose (180 mg) of brigatinib, but this case showed that symptoms can also arise after the lower dose (90 mg) (19). Moreover, given the adverse effects with the lower dose, there was a higher toxicity risk of rechallenging our patient with brigatinib in the future.

The cornerstone of treatment for symptomatic pneumonitis is corticosteroids with weight-based dosing of methylprednisolone 1–2 mg kg^−1^ day^−1^ (19). Empiric antibiotics are utilized for any suspicion of underlying infection. According to the literature, TKIs can either be cautiously continued or temporarily withheld for grade 1 or 2 pneumonitis until after the resolution of symptoms and permanently discontinued for grade 3 or 4 pneumonitis. Previously, patients have demonstrated successful rechallenging of brigatinib therapy initially dose-reduced to 30 mg daily and ramped up to 180 mg daily over 2 weeks (19). Our patient’s pneumonitis resolved with steroids and empiric antibiotics with diuretics. Brigatinib restart was deferred due to high-grade toxicity, and she responded well to treatment with alectinib.

Providers should suspect brigatinib-induced pneumonitis in patients with shortness of breath, hypoxia, and bilateral airspace disease soon after starting treatment. Our patient developed severe pneumonitis 38 h after TKI initiation, requiring intubation within 3 days. Tissue damage was rapidly reversed with high-dose corticosteroids. Despite grade 4 pneumonitis necessitating brigatinib discontinuation, her mNSCLC responded well to alectinib. The decision to start alectinib was based on the results of the ALEX study, which showed that the rate of investigator-assessed progression-free survival was significantly higher with alectinib than with crizotinib in treatment-naive ALK-positive NSCLC (20, 21). Alectinib is also generally associated with a lower incidence of pneumonitis among all ALK TKIs, a priority for our patient who had a significant pulmonary adverse event after brigatinib administration (10). Moreover, alectinib has demonstrated strong central nervous system (CNS) penetrance, comparable to that of brigatinib, and fewer serious adverse events compared with second-generation ALK TKIs, brigatinib and ceritinib, and a third-generation ALK TKI, lorlatinib (5, 22, 23). The general side effects of alectinib are limited to mild GI effects, myalgia, and leg swelling (23). Another case report highlighted ceritinib-induced pneumonitis that did not recur after transitioning to crizotinib or brigatinib, further emphasizing that the intolerability of one ALK TKI does not preclude the use of other TKIs (24). Despite the lack of standardized guidelines on ALK TKI washout periods and institutional practice differences, the team initiated alectinib relatively early on after the resolution of pneumonitis due to the high disease burden of the patient, the uniquely rapid reversal of hypoxia and pulmonary infiltrates, inpatient monitoring for immediate toxicities, and the lower pneumonitis risk of alectinib.

## Conclusion

Brigatinib is associated with the highest incidence of pulmonary toxicity compared with alternative ALK TKIs, however is widely used given its ability to overcome TKI resistance. This case underscores close monitoring of the toxicities of ALK TKIs, specifically brigatinib, for NSCLC. It also highlights the importance of considering alternative treatment options after severe adverse reactions. For our patient, subsequent management with alectinib yielded favorable disease response without recurrence of pulmonary toxicity. Although we highlighted only one case of severe brigatinib-induced pneumonitis and cannot predict the responses of other patients, collaboration between oncologists, pulmonologists, and interventionalists is essential for optimizing patient care and outcomes. Future research directions could include prospective studies to better characterize the incidence, risk factors, and underlying mechanisms of ALK TKI-induced pneumonitis, along with the development of predictive biomarkers to identify patients at higher risk of developing pulmonary toxicity. Furthermore, exploring novel treatment strategies to mitigate pulmonary adverse effects and to improve the safety profile of ALK TKIs remains an important avenue for future investigation.

## Data Availability

The original contributions presented in the study are included in the article/**Supplementary Material**. Further inquiries can be directed to the corresponding author.

## References

[1] Hallberg B Palmer RH. The role of the ALK receptor in cancer biology. Ann Oncol. (2016) 27:iii4–iii15. doi: 10.1093/annonc/mdw301 27573755

[2] Bedi S Khan SA AbuKhader MM Alam P Siddiqui NA Husain A. A comprehensive review on Brigatinib - A wonder drug for targeted cancer therapy in non-small cell lung cancer. Saudi Pharm J. (2018) 26:755–63. doi: 10.1016/j.jsps.2018.04.010 PMC6128722 30202213

[3] Jain R Chen H. Spotlight on brigatinib and its potential in the treatment of patients with metastatic ALK-positive non-small cell lung cancer who are resistant or intolerant to crizotinib. Lung Cancer (Auckl). (2017) 13:169–77. doi: 10.2147/LCTT.S126507 PMC5648304 29075144

[4] Xing P Hao X Zhang X Li J. Efficacy and safety of brigatinib in ALK-positive non-small cell lung cancer treatment: A systematic review and meta-analysis. Front Oncol. (2022) 12:920709. doi: 10.3389/fonc.2022.920709 36408160 PMC9669367

[5] Hou H Sun D Liu K Jiang M Liu D Zhu J. The safety and serious adverse events of approved ALK inhibitors in Malignancies: a meta-analysis. Cancer Manag Res. (2019) 11:4109–18. doi: 10.2147/CMAR.S190098 PMC6511621 31190983

[6] Costa RB Costa RL Talamantes SM Kaplan JB Bhave MA Rademaker A. Systematic review and meta-analysis of selected toxicities of approved ALK inhibitors in metastatic non-small cell lung cancer. Oncotarget. (2018) 9(31):22137–46. doi: 10.18632/oncotarget.25154 PMC5955140 29774128

[7] Peerzada MM Spiro TP. Pulmonary toxicities of tyrosine kinase inhibitors. Clin Adv Hematol Oncol. (2011) 9:824–36. 22252615

[8] Vahid B Marik PE. Pulmonary complications of novel antineoplastic agents for solid tumors. Chest. (2008) 133:528–38. doi: 10.1378/chest.07-0851 18252919

[9] Ng TL Narasimhan N Gupta N Venkatakrishnan K Kerstein D Camidge R. Early-onset pulmonary events associated with brigatinib use in advanced NSCLC. J Thoracic Oncol. (2020) 17:1190–9. doi: 10.1016/j.jtho.2020.02.011 32135189

[10] Qie W Zhao Q Yang L Zou B Duan Y Yao Y. Incidence of pneumonitis following the use of different anaplastic lymphoma kinase tyrosine kinase inhibitor regimens: An updated systematic review and meta-analysis. Cancer Med. (2023) 12:13873–84. doi: 10.1002/cam4.5913 PMC10358266 37017467

[11] Gettinger SN Bazhenova LA Langer CJ Salgia R Gold KA Rosell R. Activity and safety of brigatinib in ALK-rearranged non-small-cell lung cancer and other Malignancies: a single-arm, open-label, phase 1/2 trial. Lancet Oncol. (2016) 17:1683–96. doi: 10.1016/S1470-2045(16)30392-8 27836716

[12] Matsuno O. Drug-induced interstitial lung disease: mechanisms and best diagnostic approaches. Respir Res. (2012) 13:39. doi: 10.1186/1465-9921-13-39 22651223 PMC3426467

[13] Gu X Zhong Y Huang H. Case report: EGFR-TKI rechallenge after osimertinib-induced interstitial lung disease: a case report and literature review. Front Pharmacol. (2024) 15:1410684. doi: 10.3389/fphar.2024.1410684 38895622 PMC11183107

[14] Husseini KE Chaabane N Mansuet-Lupo A Leroy K Revel M Wislez M. Capmatinib-induced interstitial lung disease: A case report. Curr Problems Cancer: Case Reports. (2020) 2:100024. doi: 10.1016/j.cpccr.2020.100024

[15] Cherri S Noventa S Fanelli M Calandra G Prochilo T Bna C. Drug-related pneumonitis in cancer treatment during the COVID-19 era. Cancers (Basel). (2021) 13:1052. doi: 10.3390/cancers13051052 33801385 PMC7958630

[16] Hwang HJ Kim MY Choi CM Lee JC. Anaplastic lymphoma kinase inhibitor-related pneumonitis in patients with non-small cell lung cancer: Clinical and radiologic characteristics and risk factors. Med (Baltimore). (2019) 98:e18131. doi: 10.1097/MD.0000000000018131 PMC6890272 31770246

[17] Koshikawa K Terada J Abe M Iwasawa S Sakayori M Yoshioka K. Clinical characteristics and risk factors of drug-induced lung injury by ALK tyrosine kinase inhibitors: A single center retrospective analysis. Thorac Cancer. (2020) 11:1495–502. doi: 10.1111/1759-7714.13416 PMC7262910 32237210

[18] Dong J Li L Deng T Song H Zhang S Zhong M. Interstitial lung disease associated with ALK inhibitors and risk factors: an updated comparative pharmacovigilance analysis. Front Pharmacol. (2024) 15:1361443. doi: 10.3389/fphar.2024.1361443 39399468 PMC11466793

[19] Camidge RD Pabani A Miller RM Rizvi NA Bazhenova L. Management strategies for early-onset pulmonary events associated with brigatinib. J Thoracic Oncol. (2019) 14:1547–55. doi: 10.1016/j.jtho.2019.04.028 31108247

[20] Kim D Tiseo M Ahn M Reckamp KL Hansen KH Kim S. Brigatinib in patients with crizotinib-refractory anaplastic lymphoma kinase-positive non-small-cell lung cancer: a randomized, multicenter phase II trial. J Clin Oncol. (2017) 35:2490–8. doi: 10.1200/JCO.2016.71.5904 28475456

[21] Peters S Camidge R Shaw AT Gadgeel S Ahn JS Kim D. Alectinib versus crizotinib in untreated ALK-positive non–small-cell lung cancer. NEJM. (2017) 377:829–38. doi: 10.1056/NEJMoa1704795 28586279

[22] Gil M Knetki-Wróblewska M Niziński P Strzemski M Krawczyk P. Effectiveness of ALK inhibitors in treatment of CNS metastases in NSCLC patients. Ann Med. (2023) 55:1018–28. doi: 10.1080/07853890.2023.2187077 PMC10795653 36896848

[23] Ando K Manabe R Kishino Y Kusumoto S Yamaoka T Tanaka A. Comparative efficacy and safety of lorlatinib and alectinib for ALK-rearrangement positive advanced non-small cell lung cancer in Asian and non-Asian patients: A systematic review and network meta-analysis. Cancers (Basel). (2021) 13:3704. doi: 10.3390/cancers13153704 34359604 PMC8345181

[24] Bender L Meyer G Quoix E Mennecier B. Ceritinib-related interstitial lung disease improving after treatment cessation without recurrence under either crizotinib or brigatinib: a case report. Ann Transl Med. (2019) 7:106. doi: 10.21037/atm.2019.01.24 31019956 PMC6462648

